# Heart rhythm complexity impairment in patients undergoing peritoneal dialysis

**DOI:** 10.1038/srep28202

**Published:** 2016-06-21

**Authors:** Yen-Hung Lin, Chen Lin, Yi-Heng Ho, Vin-Cent Wu, Men-Tzung Lo, Kuan-Yu Hung, Li-Yu Daisy Liu, Lian-Yu Lin, Jenq-Wen Huang, Chung-Kang Peng

**Affiliations:** 1 Department of Internal Medicine, National Taiwan University Hospital and National Taiwan University College of Medicine, Taipei, Taiwan; 2 Biomedical Engineering, National Taiwan University Hospital and National Taiwan University College of Medicine, Taipei, Taiwan; 3 Department of Biomedical Sciences and Engineering, National Central University, Chungli, Taiwan; 4 Graduate Institute of Biomedical Electronics and Bioinformatics, National Taiwan University, Taipei, Taiwan; 5 Department of Agronomy, Biometry Division, National Taiwan University, Taipei, Taiwan; 6 Division of Interdisciplinary Medicine and Biotechnology, Beth Israel Deaconess Medical Center/Harvard Medical School, Boston, Massachusetts, USA

## Abstract

Cardiovascular disease is one of the leading causes of death in patients with advanced renal disease. The objective of this study was to investigate impairments in heart rhythm complexity in patients with end-stage renal disease. We prospectively analyzed 65 patients undergoing peritoneal dialysis (PD) without prior cardiovascular disease and 72 individuals with normal renal function as the control group. Heart rhythm analysis including complexity analysis by including detrended fractal analysis (DFA) and multiscale entropy (MSE) were performed. In linear analysis, the PD patients had a significantly lower standard deviation of normal RR intervals (SDRR) and percentage of absolute differences in normal RR intervals greater than 20 ms (pNN20). Of the nonlinear analysis indicators, scale 5, area under the MSE curve for scale 1 to 5 (area 1–5) and 6 to 20 (area 6–20) were significantly lower than those in the control group. In DFA anaylsis, both DFA α1 and DFA α2 were comparable in both groups. In receiver operating characteristic curve analysis, scale 5 had the greatest discriminatory power for two groups. In both net reclassification improvement model and integrated discrimination improvement models, MSE parameters significantly improved the discriminatory power of SDRR, pNN20, and pNN50. In conclusion, PD patients had worse cardiac complexity parameters. MSE parameters are useful to discriminate PD patients from patients with normal renal function.

Cardiovascular disease is the leading cause of mortality and morbidity in patients with chronic kidney disease or end-stage renal disease (ESRD) undergoing either hemodialysis or peritoneal dialysis (PD)^1^,^2^. Sudden cardiac death is one of the most common causes of mortality, with an estimated rate of around 7% per year in patients with ESRD^3^. One explanation for this high rate is autonomic nervous system dysfunction^3^,^4^. Both parasympathetic damage and sympathetic nerve overactivity are common in ESRD patients^5^. Furthermore, sympathetic nerve overactivity is associated with mortality and cardiovascular outcomes in ESRD patients^6^.

Analysis of variations in heart rate oscillation, commonly known as heart rate variability (HRV), is commonly used to assess autonomic function in clinical studies due to its simple and noninvasive approach^7^. Initially, HRV was derived by linear methods such as Fourier transform by using certain frequency ranges to represent sympathetic and parasympathetic activities^7^. This concept has been proved by one large scale study showing that HRV parameters from linear method could predict outcomes in patients with cardiovascular disease^8^. In recent years, newer methods based on nonlinear and nonstationary signal modeling have been developed and successfully applied^9^. The concept of heart rhythm complexity analysis using nonlinear methods including detrended fractal analysis (DFA) or multiscale entropy (MSE) is based on the assumption that a healthy system exerts meaningful complex control over time to maintain operation in an ever-changing environment^10^,^11^. Conversely, decreased complexity of heart rate dynamics has been demonstrated in patients with various diseases such as heart failure, stroke, sepsis, primary aldosteronism and critical illnesses requiring extracorporeal life support^12^,^13^,^14^,^15^,^16^. Compared to traditional HRV parameters based on linear methodology, heart rhythm complexity analysis has been shown to have a better predictive power for prognosis in patients with cardiovascular disease^16^,^17^.

Only few studies evaluate heart rhythm complexity in ESRD patients^18^,^19^,^20^. All these studies have used DFA or approximate entropy anaylsis to derive heart rhythm complexity^19^,^20^. So far, no study has reported the results of MSE analysis in ESRD patients. Besides, most of the studies evaluate the prognostic prediction in ESRD patients without enrolling control groups. Since renal impairment might have direct or indirect effect (through cardiovascular disease) on autonomic nervous system, in this study, we plan to enroll a group of ESRD patients without cardiovascular disease and a group of subjects with normal renal function to evaluate the direct effect of ESRD on heart rhythm complexity.

## Results

### Patients

Sixty-five patients (39 men) undergoing PD without prior cardiovascular disease and 72 individuals (36 men) with normal renal function (control group) were enrolled in this study. The reason of PD implementation were variable primary glomerulonephritis in 47 patients , diabetic nephropathy in 9 patients, lupus nephritis in 3 patients, polycystic kidney disease in 2 patients and other causes in 4 patients.

The clinical data are shown in Table 1. The PD patients had significantly higher rates of using beta-blockers and calcium channel blockers than the controls. In addition, the PD patients had higher levels of serum fasting glucose and creatinine, and lower levels of sodium and potassium than the controls. The left ventricular ejection fraction was comparable in both groups.

### Holter data

In linear analysis, the PD patients had a significantly lower standard deviation of normal RR intervals (SDRR), percentage of absolute differences in normal RR intervals greater than 20 ms (pNN20), the percentage of absolute differences in normal RR intervals greater than 50 ms (pNN50), low frequency (LF), high frequency (HF), and low/high frequency ratio than control participants (Table 2).

**Figure 1:**
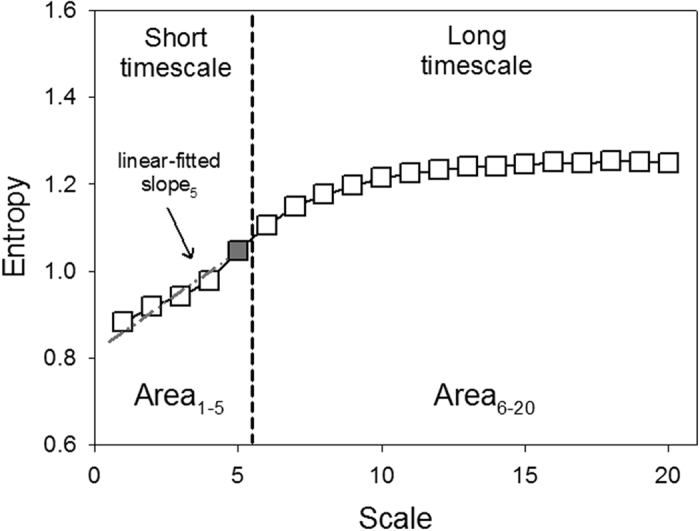
Quantification of MSE: Summation of the entropy over different scales can quantify the complexity over certain timescales. Four parameters of the MSE were assessed. The first was the linear-fitted slope between scales 1–5 (slope 1–5). The second was the entropy value of scale 5 (scale 5). The area under the curve between scale 1–5 (area 1–5) was used to represent complexity between short scales. For longer scales, the common profile of entropy gradually increased as the time scale increased and reached a plateau where information richness could be accumulated rapidly if the system responded well. We used the area under curve between scale 6–20 (area 6–20) to represent complexity between long scales.

**Figure 2:**
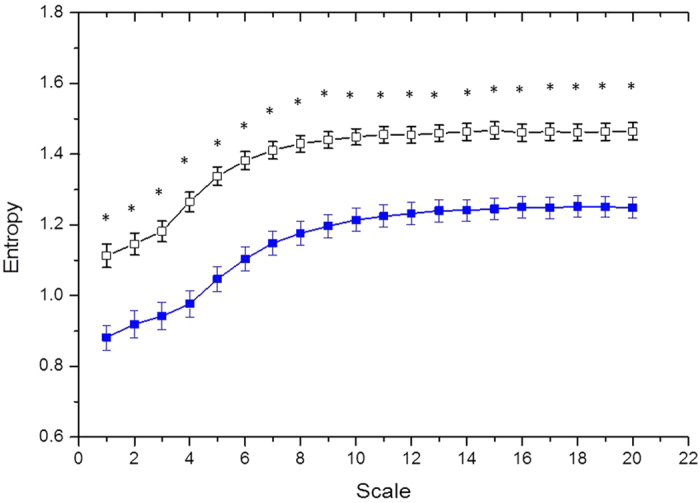
The entropy over different time scales in PD (blue solid square box) patients and patients with normal renal function (black empty square box). *p < 0.001.

In DFA anaylsis, both DFA α1 and DFA α2 were comparable in both groups. The quantification of MSE parameters is shown in Fig. 1. The entropy over different time scales in the two group of patients is shown in Fig. 2. The PD patients had significant lower entropy in each time scale than control group . Of the nonlinear analysis indicators, scale 5, area under the MSE curve for scale 1 to 5 (area 1–5) and 6 to 20 (area 6–20) were significantly lower than those of the control group.

### Correlation between linear and MSE parameters

In analysis of all participants, scale 5, area 1–5 and area 6–20 were significantly correlated with both time and frequency domain parameters (Table 3). In contrast, slope 1–5 was only significantly correlated with frequency but not time domain parameters. In subgroup analysis, short time scales complexity parameters (scale 5 and area 1–5) were significantly correlated with pNN20 and HF in both groups. Long time scales complexity parameters (area 6–20) were also significantly correlated with very low frequency (VLF), LF and pNN20 in both groups. There were some differences in correlation patterns between the PD and control groups.

### Comparisons of all linear and nonlinear parameters to differentiate the two groups

**Figure 3:**
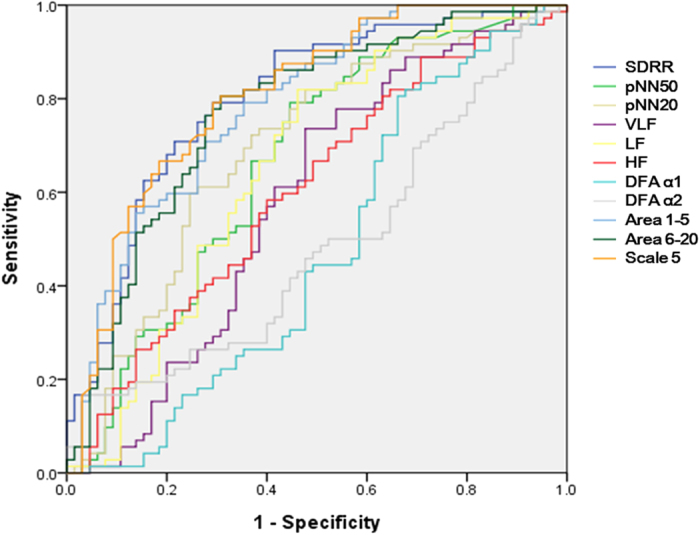
Analysis of the discrimination power of the two group by receiver operating characteristic curve analysis. The areas under the curve of SDRR, pNN50, pNN20, VLF, LF, HF, DFAα1, DFAα2, slope 1–5, area 1–5, area 6–20, and scale 5 were 0.800, 0.667, 0.693, 0.584, 0.657, 0.603,0.471,0.485, 0.582, 0.786, 0.771, and 0.806, respectively.

In receiver operating characteristic (ROC) curve analysis, scale 5 had the greatest discriminatory power for the two groups compared to all other linear and non-linear parameters (Fig. 3). The areas under the curve (AUC) of scale 5, SDRR, pNN50, PNN20, VLF, LF, HF, DFA α1, DFA α2, slope 1 to 5, area 1–5, area 6–20 were 0.806, 0.800, 0.667, 0.693, 0.584, 0.657, 0.603, 0.471, 0.485, 0.582, 0.786 and 0.771, respectively.

### The advantage of adding MSE parameters to linear parameters to discriminate the two groups

In both net reclassification improvement (NRI) and integrated discrimination improvement (IDI) models, three MSE parameters (scale 5, area 1–5, and area 6–20) significantly improved the discriminatory power of SDRR, pNN20, and pNN50 (Table 4). Scale 5 was especially good in the models for pNN20 and pNN50.

## Discussion

The major findings of this study were: 1) the PD patients had worse heart rhythm complexity than those with normal renal function; 2) in all linear and non-linear parameters, scale 5 and SDRR had the greatest single discriminatory power to detect the patients undergoing PD; 3) the combination of linear and non-linear improved the discriminatory power to differentiate PD patients from patients with normal renal function.

Traditional linear analysis of HRV is a useful tool to evaluate the autonomic system, and is commonly used to stratify the risk of patients with cardiovascular disease^8^,^21^. ESRD is characterized by a high prevalence of sudden cardiac death and autonomic nervous system dysfunction including parasympathetic damage and sympathetic nerve overactivity^3^,^4^,^5^. Therefore, HRV is also a useful tool to predict mortality and cardiovascular outcomes in ESRD patients^22^,^23^. In our results, the PD patients had significantly lower values of several linear parameters, which again reflect prominent autonomic dysfunction in ESRD patients.

DFA is a scaling analysis method to represent the correlation property of a signal. The physiological background of DFA has been demonstrated to be associated with a delicate interplay between sympathetic and vagal outflow^24^. The breakdown this correlation property occur only when sympathetic and vagal outflow are co-activated. For patients with ESRD, Suzuki *et al.* demonstrated the superiority of DFA analysis compared to traditional linear parameters to predict 5-year mortality rates in hemodialysis patients^18^. Furthermore, in the same study, the addition of scaling exponent α1 (a DFA parameter) to the clinical risk factors significantly improved the prediction of mortality. Although DFA is a predictor for clinical outcomes, in the current study, we showed that PD patients had comparable DFA parameters to the control patients. To our knowledge, no previous study has compared DFA parameters between patients with ESRD and those with normal renal function. Perhaps, ESRD patients without cardiovascular disease still maintain a certain degree of normal interaction (no co-activation) and thus preserve the nonlinear scaling behavior of heart rate dynamics.

Complexity is a concept that lies between periodicity and randomness. Heart rate dynamics is a complex system. The nonlinear methods viewed the healthy heart beat fluctuation as an output of an integrative control system with multiple interacting physiological processes operating at different time scales which emerge as complex dynamical patterns. When disease status developed, the system breakdown into either periodic-like^14^,^16^ (for example, heart failure) or random-like status^25^ (for example, atrial fibrillation). Recently, the MSE method, which was specifically developed to treat heterogeneous complexity, has shown the ability to extend the traditional entropy algorithm to quantify information richness over multiple time scales in physiological systems^26^. Slope 1–5, area 1–5, and scale 5 of MSE were the quantitative estimation of information richness over short timescales. The slope 1–5 may outline the structure of heart rate dynamics and negative slope was observed in patients with heart failure, atrial fibrillation or critical illness, indicating highly irregular but less information richness structure (i.e. uncorrelated fluctuations with the loss of feedback interactions)^14^,^26^. On the contrary, when the RR intervals of the individuals were entrained by respiration, the higher the respiratory modulated amplitude, the lower the entropy value expected. Since coarse-graining procedure over small timescales filtered out the periodic respiratory oscillations^26^, the sample entropies of heartbeat fluctuations increased over short timescales and slope 1–5, therefore, exhibited positive value. The area 1–5 in MSE probes the complexity structure of the heart rate dynamics and the scale 5 may give a powerful overall combined estimation of heart rate short-term complexity and the integrity of sinus arrhythmia^26^. The indexes of long-term scales were more controversial since several physiological mechanisms beneath the time scales. The important physiological mechanisms such as baroreflex and hormonal system can be the contributing factors^15^. For example, excess aldosterone not only cause cardiovascular damage^27^,^28^,^29^, but impaired both short-term and long-term scales of MSE^15^.

The usefulness of MSE is not limited to the cardiovascular system, and it has also been shown to be able to predict the outcomes of patients with severe trauma requiring treatment in an intensive care unit and across the diverse spectrum of traumatic injury^30^, the neurological outcomes of patients after stroke^12^, and the clinical consequences of sepsis^13^. However, no previous study has reported the results of MSE analysis in ESRD patients. In the present study, we found that PD patients had significantly decreased MSE parameters such as scale 5, area 1–5, and area 6–20 and these parameters significantly improved the predictive power of linear parameters. This demonstrates the additive effects of linear and non-linear parameters to differentiate PD patients from patients with normal renal function.

In the present study, scale 5 had the best discriminatory power in both groups. Similar findings have also been reported in heart failure and older patients^11^. Costa *et al.* found the largest separation between heart failure patients and healthy subjects is obtained for scale 5. Interesting, the strongest separation was also obtained for scale 5 for separation from elder from young subjects^11^. Taken together with our findings, this implies that scale 5 may be a good marker to detect a loss of complexity by age or disease status, although the underling mechanism is unclear. However, a marker that can differentiate patients from healthy participants may not be the same as a marker that can predict prognosis. For example, in heart failure patients, instead of short time scale parameter, long time scale parameters (area 6–20) have been shown to have the best prognostic predictive power^16^. Further studies are needed to elucidate whether scale 5 is also a prognosis marker in PD patients.

Patients with ESRD carry a higher risk of developing cardiovascular disease including vasculopathy and cardiomyopathy secondary to pressure and volume overload^1^,^2^,^3^. These could cause a loss of complexity in heart rate dynamics in patients with ESRD. However, in the present study, we enrolled patients without obvious cardiovascular disease therefore, the mechanism leading to deterioration of MSE are not secondary to it. Furthermore, we enrolled PD patients since the fluid status of hemodialysis patients vary a lot day by day and might interfere the measurements of HRV^28^. Even though the direct effect of uremic on autonomic nervous function is not clear, uremic toxins with a molecular weight of 300–12000, so called middle molecules were suggested to be the major etiology^31^. Studies also demonstrated that PD and hemodialysis with dialyzer membrane highly permeable to middle molecules could dramatically reduce the prevalent of uremic neuropathy^32^.

There are several limitations to this study. First, we only enrolled PD patients, and further studies are needed to validate whether our findings can be applied to hemodialysis patients. Second, this cross-sectional study aimed to investigate impairments in heart rhythm complexity in ESRD patients, and whether MSE parameters can predict the clinical prognosis is unclear.

In conclusion, the PD patients had impaired cardiac complexity. Scale 5 in MSE studies had the greatest discriminatory power to differentiate PD patients from patients with normal renal function. In addition, MSE parameters significantly improved the discriminatory power of linear parameters.

## Methods

### Patients

In this prospective, cross-sectional study, we enrolled 65 Taiwanese patients who received PD with conventional glucose-based lactate-buffered solutions (UltraBag; Baxter Healthcare SA, Singapore) and 72 participants (control group) who visited for health check-up at National Taiwan University Hospital.

The PD patients were enrolled prospectively for a cohort study. Some study results regarding echocardiography or laboratory data were published^33^,^34^. The inclusion criteria for patients group in this study were 1) received PD for more than 6 months; 2) no history of cardiovascular disease such as atrial fibrillation, significant valvular heart disease, coronary artery disease, myocardial infarction, heart failure, cerebrovascular events, or peripheral artery disease.

The participants in control group were prospectively enrolled for this study. The inclusion criteria were 1) estimated glomerular filtration rate >60 ml/min according to the Chinese Modification of Diet in Renal Disease Study equation^35^; 2) no history of cardiovascular disease such as atrial fibrillation, significant valvular heart disease, coronary artery disease, myocardial infarction, heart failure, cerebrovascular events, or peripheral artery disease.

A medical history of each participant including demographics and medications was carefully recorded and biochemical parameters were measured at the first evaluation. All patients underwent 24-h ambulatory ECG Holter recording (ZymedDigiTrak Plus 24-Hour Holter Monitor Recorder and Digitrak XT Holter Recorder 24 Hour, Philips, Amsterdam, Netherlands). Standard transthoracic echocardiography (iE33 xMATRIX Echocardiography System, Philips, Amsterdam, Netherlands) was performed in each patient. This study was approved by the Institutional Review Board of National Taiwan University Hospital, and all subjects provided written informed consent including for storage of their information in the hospital database and usage for research. The methods in the study were carried out in accordance with the approved guidelines.

### Data pre-processing

An epoch of four hours of daytime RR intervals (between 9AM and 5PM) was selected for analysis^36^. The selected ECGs were automatically annotated using an automatic algorithm and carefully corrected by an experience technician.

### Time and frequency domain analysis

SDRR was calculated and taken to represent the overall variability of autonomic modulation. Two pNNx parameters (pNN50 and pNN20) were calculated as the percentage of absolute differences in normal RR intervals greater than x ms. The threshold of x ms can filter out the RR changes with larger amplitudes, and better evaluate the function of the autonomic system^37^. The frequency domain parameters, high frequency (HF; 0.15–0.4 Hz), low frequency (LF; 0.04–0.15Hz), and very low frequency (VLF; 0.003–0.04) power, were also calculated by fast Fourier transformation according to the recommendations of the European Society of Cardiology and the North American Society of Pacing Electrophysiology^7^.

### Nonlinear methods

The parameters derived from nonlinear methods were used to quantify the important characteristics of the physiological systems beyond variability such as scale-invariant (fractal behavior) underlying the signals that vary with time^26^,^38^.

### DFA analysis

DFA can be used to evaluate the fractal behavior beneath the RR dynamics originating from well-regulated, interconnected systems^38^. The external trends related to environmental interference were fit linearly over different scales (e.g. 1, 2, 3… and n beats) and removed from the integrated time series. Fluctuations in each scale were calculated by summing up the detrended integrated time series. The slope (α exponent) of the logarithmic plot of fluctuations against time scales were computed and taken to indicate the fractal correlation property of the time series.

Short-term RR intervals are predominately modulated by respiratory sinus arrhythmia in normal subjects causing drastic changes in the slope between short- and long-term timescales. Crossover can be quantified by the α exponents of RR dynamics over short (α1; 4–11 beats) and long (α2; 11–64 beats) timescales to better probe the fractal property of the physiological system^38^.

### MSE analysis

Instead of merely estimating the predictability of a time series with a single scale, MSE provides meaningful information richness embedded in different timescales by the degree of predictable sequential changes over the timescale^26^. The time series of different time scales were reconstructed using a coarse-graining process (i.e. averaging the non-overlapping n consecutive beats to form the new time series) and quantified by sample entropy^39^. The calculated entropy can then be used to represent the function of scale to assess the complex structure of the physiological signals, and the profile of the MSE curve can be used to assist the clinical categorization of several diseases^26^. In this study, four different parameters were calculated from the MSE profile: the entropy value of scale 5 (scale 5), the summation of entropy values of scales 1–5 (area 1–5) or 6–20 (area 6–20) to quantify the complexity of RR dynamics exhibited in short and long timescales. The linear-fitted slope of scale 1–5 (slope 5) was also calculated to characterize the regulatory behavior of the underlying system over a short timescale (Fig. 1).

To avoid underestimation of entropy due to external non-stationarity, the RR dynamics were detrended by removing any trend longer than 2 hours before performing MSE. The empirical mode decomposition (EMD) method, which is based on Hilbert-Huang transformation, was used to adaptively extract the trends and subtract them from the original RR interval signals^40^, since EMD algorithm can better approximate hidden trends in complex time series^40^,^41^,^42^.

### Echocardiography

Standard transthoracic echocardiography (iE33 xMATRIX Echocardiography System, Philips, Amsterdam, Netherlands) was performed in each patient. The echocardiographic measurements included two-dimensional, M-mode and Doppler ultrasound recordings. Left ventricular dimension, interventricular septum and posterior wall thicknesses, and left ventricular ejection fraction (M-mode) were measured via a parasternal long axis view.

### Statistical analysis

Continuous variables were presented as median (25^th^–75^th^ percentile). Comparisons of continuous data between the PD and control groups were made using the Mann-Whitney U-test. Differences between proportions were assessed using the chi-square test or Fisher’s exact test. Correlation tests were performed using Spearman’s correlation tests.

In order to compare the ability of different Holter parameters to differentiate the PD patients from the control patients, we used area under the ROC curve analysis with a logistic regression models. We used C-statistics to describe the discrimination of the models before and after adding non-linear parameters^43^,^44^,^45^.

NRI and IDI models were used to assess improvements in prediction using two different logistic regression models^44^, with 0.2 and 0.4 used as the cutoff points. NRI is equal to sum of the increasing probability for survivors and decreasing probability for non-survivors subtracted by the decreasing probability for and increasing probability for non-survivors after adopting the updated model. IDI is defined as the average improvement of survival probability for all patients after adopting the updated model. All statistical analyses were performed using R software (http://www.r-project.org/), version 2.15.2. Statistical significance was set at p < 0.05.

## Additional Information

**How to cite this article**: Lin, Y.-H. *et al.* Heart rhythm complexity impairment in patients undergoing peritoneal dialysis. *Sci. Rep.* **6**, 28202; doi: 10.1038/srep28202 (2016).

## Figures and Tables

**Table 1:** Clinical data of the patients.

	Controls N = 72	Patients undergoing peritoneal dialysis N = 65	*p* value
Mean age (years)	55 (45–61)	56 (48–63)	0.661
Male, n (%)	36 (50)	39 (60)	0.303
Diabetes mellitus, n (%)	13 (18)	8 (12)	0.477
Hypertension, n (%)	52 (79)	58 (89)	0.161
Medication, n (%)			
ACEI or ARB	33 (46)	31 (48)	0.865
Beta-blocker	28 (39)	42 (65)	0.003
CCB	37 (51)	45 (69)	0.038
Glucose, mg/dL	94 (88–106)	104 (92–130)	0.005
Creatinine, mg/dL	0.9 (0.7–1.0)	11.1 (9.2–12.9)	<0.001
Triglyceride, mg/dL	122 (81–165)	150 (97–234)	0.052
Total cholesterol, mg/dL	193 (171–211)	189 (159–226)	0.943
Na, mmol/L	139 (138–141)	136 (133–138)	<0.001
K, mmol/L	4.2 (3.9–4.4)	4.0 (3.2–4.3)	0.005
Ca, mmol/L	9.3 (9.0–9.7)	9.8 (8.6–10.3)	0.064
LVEF, %	70 (67–74)	68 (64–73)	0.215

Data were presented as median (25^th^–75^th^ percentile) or number (percentage). CCB = calcium channel blocker; ACE-I = angiotensin converting enzyme inhibitor; ARB = angiotensin receptor blocker; LVEF = left ventricular ejection fraction.

**Table 2:** Holter parameters of the patients.

	Controls N = 72	Patients undergoing peritoneal dialysis N = 65	*p* value
Time domain analysis			
Mean RR, ms	771 (677; 850)	799 (731–895)	0.149
SDRR, ms	76.8 (62.6–93.2)	44.1 (30.3–65.5)	<0.001
pNN50, %	2.04 (0.62–4.96)	0.53 (0.08–3.19)	0.001
pNN20, %	20.2 (9.9–33.9)	7.51 (2.74–18.51)	<0.001
Frequency domain analysis			
Very low frequency	931 (689–1365)	713 (431–1424)	0.092
Low frequency	261 (171–452)	153 (62–370)	0.001
High frequency	87 (44–166)	55 (31–120)	0.037
Low/high frequency ratio	3.17 (1.89–4.90)	2.31 (1.31–3.60)	0.013
Detrended fluctuation analysis			
α1	1.14 (1.09–1.30)	1.17 (1.01–1.37)	0.552
α2	1.19 (1.13–1.28)	1.20 (1.13–1.28)	0.760
Multiscale entropy			
Slope 1–5	0.059 (0.011–0.103)	0.043 (0.009–0.071)	0.098
Scale 5	2.75 (2.40–3.01)	2.06 (1.77–2.48)	<0.001
Area 1–5	6.06 (5.16–6.64)	4.70 (3.87–5.54)	<0.001
Area 6–20	22.29 (19.90–23.61)	18.2 (15.9–20.9)	<0.001

Data were presented as median (25^th^–75^th^ percentile). SDNN = standard deviation of normal RR intervals; pNN20 = percentage of the absolute change in consecutive normal RR interval exceeds 20 ms; pNN50 = percentage of the absolute change in consecutive normal RR interval exceeds 50.

**Table 3:** Correlation between linear and MSE parameters.

	Total (n = 137)				PD (n = 65)				Control (n = 72)			
	Slope 1–5	Scale 5	Area 1–5	Area 6–20	Slope 1–5	Scale 5	Area 1–5	Area 6–20	Slope 1–5	Scale 5	Area 1–5	Area 6–20
SDRR, ms	0.078	0.313*	0.320*	0.210^#^	0.234	0.141	0.081	0.122	−0.122	−0.044	0.048	−0.145
pNN50, %	−0.136	0.367*	0.480*	0.192^#^	−0.044	0.189	0.262^#^	0.046	−0.340^¥^	0.356^¥^	0.529*	0.144
pNN20, %	−0.129	0.491*	0.604*	0.317*	0.017	0.408*	0.452*	0.272^#^	−0.425*	0.369*	0.593*	0.161
Very low frequency	0.245^¥^	0.294*	0.223^¥^	0.247^¥^	0.413*	0.217	0.044	0.248^#^	0.100	0.323^¥^	0.322^¥^	0.254^#^
Low frequency	0.388*	0.533*	0.448*	0.433*	0.535*	0.397*	0.225	0.402*	0.254^#^	0.517*	0.465*	0.335^¥^
High frequency	−0.125	0.363*	0.477*	0.204^#^	0.064	0.269^#^	0.301^#^	0.176	−0.324^¥^	0.383*	0.581*	0.165

Values are correlation coefficients; *p < = 0.001; ^¥^p < 0.01; ^#^p < 0.05. SDRR = standard deviation of normal RR intervals; pNN20 = percentage of the absolute change in consecutive normal RR interval exceeds 20 ms; pNN50 = percentage of the absolute change in consecutive normal RR interval exceeds 50 ms.

**Table 4:** AUC, NRI, and IDI models of linear parameters before and after adding MSE parameters.

Parameters		AUC	R square	NRI	NRI P-value	IDI	IDI P-value
SDRR		0.800	0.255				
	Area1 to 5	0.853	0.358	0.635	<0.001	0.113	<0.001
	Area 6 to 20	0.864	0.400	0.866	<0.001	0.150	<0.001
	Scale 5	0.861	0.391	0.863	<0.001	0.144	<0.001
pNN20		0.693	0.059				
	Area1 to 5	0.786	0.205	0.623	<0.001	0.161	<0.001
	Area 6 to 20	0.784	0.230	0.915	<0.001	0.174	<0.001
	Scale 5	0.807	0.245	0.857	<0.001	0.202	<0.001
pNN50		0.667	0.000				
	Area1 to 5	0.789	0.228	0.774	<0.001	0.242	<0.001
	Area 6 to 20	0.770	0.212	0.918	<0.001	0.221	<0.001
	Scale 5	0.801	0.256	0.949	<0.001	0.269	<0.001

SRR = standard deviation of normal RR intervals; pNN20 = percentage of the absolute change in consecutive normal RR interval exceeds 20 ms; pNN50 = percentage of the absolute change in consecutive normal RR interval exceeds 50 ms; AUC: areas under the curve; NRI: net reclassification improvement; IDI: integrated discrimination improvement; MSE: multiscale entrop.
